# Extracorporeal therapies in pediatric severe sepsis: findings from the pediatric health-care information system

**DOI:** 10.1186/s13054-015-1105-4

**Published:** 2015-11-10

**Authors:** Amanda Ruth, Courtney E. McCracken, James D. Fortenberry, Kiran B. Hebbar

**Affiliations:** Division of Pediatric Critical Care Medicine, Emory University School of Medicine, 1405 Clifton Road NE, Atlanta, GA 30322 USA; Department of Pediatrics, Emory University School of Medicine, 2015 Uppergate Drive, Atlanta, GA 30322 USA; Children’s Healthcare of Atlanta, 1405 Clifton Road NE, Atlanta, GA 30322 USA

## Abstract

**Introduction:**

Pediatric severe sepsis (PSS) continues to be a major health problem. Extracorporeal therapies (ETs), defined as extracorporeal membrane oxygenation (ECMO) and RRenal replacement therapyenal replacement therapy (RRT), are becoming more available for utilization in a variety of health conditions. We aim to describe (1) rates of utilization of ET in PSS, (2) outcomes for PSS patients receiving ET, and (3) epidemiologic characteristics of patients receiving ET.

**Methods:**

We conducted a retrospective review of a prospectively collected database. Data from the Pediatric Health Information System (PHIS) database collected by the Children’s Hospital Association (CHA) from 2004–2012 from 43 US children’s hospitals’ pediatric intensive care units (PICUs) were used. Patients with PSS were defined by (1) International Classification of Diseases, 9th Revision (ICD-9) codes reflecting severe sepsis and septic shock and (2) ICD-9 codes of infection and organ dysfunction as defined by updated Angus criteria. Among the patients with PSS, those with a PHIS flag of ECMO or RRT were identified further as our main cohort.

**Results:**

From 2004 to 2012, 636,842 patients were identified from 43 hospitals, and PSS prevalence was 7.7 % (49,153 patients). Nine point eight percent (4795 patients) received at least one form of ET, and the associated mortality rate was 39 %. Mortality rates were 47.8 % for those who received ECMO, 32.3 % in RRT, and 58.0 % in RRT + ECMO. Underlying co-morbidities were found in 3745 patients (78.1 %) who received ET (81 % for ECMO, 77.9 % in RRT, and 71.2 % in those who received both). There was a statistically significant increase in ECMO utilization in patients with at least three organ dysfunctions from 2004 to 2012 (6.9 % versus 10.3 %, *P* < 0.001) while RRT use declined (24.5 % versus 13.2 %, *P* < 0.001). After 2009, there was a significant increase in ECMO utilization (3.6 % in 2004–2008 versus 4.0 % in 2009–2012, *P* = 0.004). ECMO and RRT were used simultaneously in only 500 patients with PSS (1 %).

**Conclusions:**

ETs were used in a significant portion of PSS patients with multiple organ dysfunction syndrome (MODS) during this time period. Mortality was significant and increased with increasing organ failure. ECMO use in PSS patients with MODS increased from 2004 to 2012. Further evaluation of ET use in PSS is warranted.

**Electronic supplementary material:**

The online version of this article (doi:10.1186/s13054-015-1105-4) contains supplementary material, which is available to authorized users.

## Introduction

Since their inception, extracorporeal therapies (ETs) such as extracorporeal membrane oxygenation (ECMO) and renal replacement therapies (RRTs) have become more widely available worldwide. ECMO for pediatric acute respiratory distress syndrome [1] and RRT for renal failure have become standards of treatment over the past decade. However, current national utilization rates of these technologies in cases of pediatric septic shock (PSS) across US children’s hospitals are still largely unknown.

PSS continues to be a leading cause of mortality and morbidity in children [2, 3]. In recent years, there have been significant strides made in sepsis treatment such as the advent of goal-directed therapy [4] and the consensus treatment guidelines published by the American College of Critical Medicine (ACCM) [5]. Whereas adult studies have suggested variable outcomes for ECMO in septic shock with survival rates ranging from 15 % to 70 % [6, 7], pediatric studies have been more promising. A 2011 review of Extracorporeal Life Support Organization data [8] suggested that survival rates have significantly increased in cases of PSS as compared with initial reports (an overall survival rate of 68 % versus 38.6 %) [9]. Single-center experience suggests more satisfactory outcomes in refractory PSS [10, 11]. The ACCM pediatric sepsis guidelines were also revised in 2009 to recommend consideration of ECMO in the event of refractory septic shock [12].

Likewise, there are data that suggest potential benefits of RRT use in certain populations [13]. Positive fluid balance in critically ill children has been found to be an independent risk factor for mortality in several studies, and early institution of RRT is associated with an improved outcome [14].

The objectives of this study are (a) to report utilization rate of these technologies in patients with PSS in US pediatric hospitals from 2004 to 2012, (b) analyze the demographics and clinical characteristics of PSS patients in whom ET was used, and (c) report outcomes and trends in this particular PSS cohort.

## Methods

### Data collection

This was an observational, retrospective cohort review of a prospectively collected research database. The study was approved by institutional review boards from the Children’s Hospital Association (CHA) (Overland Park, KS, USA) and Children’s Healthcare of Atlanta. Requirement for informed consent was waived. All patient-related data were de-identified prior to review and enrollment.

Data were obtained from the Pediatric Health Information System (PHIS) database, maintained by CHA, which contains demographic, outcome, and resource utilization data from 43 freestanding tertiary care children’s hospitals (Additional file 1). Participating hospitals are located in non-competing markets of 27 states plus the District of Columbia and accounted for 15 % of all pediatric hospitalizations in the US. The 43 hospitals included in PHIS provide discharge data, including patient demographics, diagnoses, and procedures. Billing data include medications, radiologic imaging studies, laboratory tests, and supplies charged to each patient. Data are de-identified prior to inclusion in the database; however, encrypted medical record numbers allow tracking of individual patients across admissions. CHA and participating hospitals jointly ensure data integrity as previously described [15, 16].

All children (0–18 years of age) admitted to a pediatric intensive care unit (PICU) in the 43 participating hospitals from January 2004 to December 2012 were identified through an ICU admission flag in PHIS. We defined PSS if patients met any of these criteria: (a) ICD-9 codes for severe sepsis (995.92) or septic shock (785.52), (b) ICD-9 codes for infection and acute organ dysfunction (OD) as previously defined by Angus et al. [17] and with codes updated by Weiss et al. [18]. Those receiving ET were further identified by individual flags for ECMO and RRT, respectively. ECMO use was defined in PHIS by an ICD procedure code of 39.65 or a clinical transaction code (CTC) of 521181. RRT use was defined in PHIS as a CTC of 525201 or 525221 [19]. Patients who had been admitted to the neonatal intensive care unit (NICU) during the same hospitalization (i.e., NICU patients subsequently transferred to PICU) were excluded as PHIS does not have the ability to distinguish whether a PSS episode or ET use occurred in the NICU or the PICU.

Analysis was performed on data from all 43 participating children’s hospitals. For purposes of trend analysis, we identified 33 hospitals that were CHA members and contributed continuous data from the years 2004 to 2012.

Demographic data were reported on the basis of their inclusion in the PHIS database. Age was divided into grouping consistent with the US National Census Bureau reporting. Underlying patient disease co-morbidities were determined by using the definition of a Pediatric Complex Chronic Condition [20]. Length of stay (LOS) was reported as median with interquartile range (IQR) being 25th and 75th percentiles. Hospitalization charges were supplied by CHA as the total amount that was charged to the patient by individual hospitals. Cost was estimated by multiplying the total hospital charge by the hospital-specific ratio of cost to charge. All reported figures were adjusted for inflation to the year 2012 by using published US Bureau of Labor Statistics data for medical cost inflation.

### Statistical analysis

Statistical analyses were performed by using SAS 9.3 (SAS Institute Inc., Cary, NC, USA). Statistical significance was assessed at the 0.05 level unless otherwise noted. Descriptive statistics were calculated for all variables of interest. Chi-squared tests were used to compare categorical variables, and two-sample *t* tests or Wilcoxon rank-sum tests were used to compare continuous variables between two groups or years. Although it was possible for a patient to have multiple PICU admissions for severe sepsis over the 9-year cohort, such patients could not be uniquely identified in the PHIS database. Thus, multiple PICU admissions were treated as independent for the purposes of statistical analyses. A generalized linear mixed model was used to assess the effect of time on ET use in patients with PSS while adjusting for OD and PSS cases clustered within hospitals. This model adjusts for the correlation among PSS cases from the same hospital. Only hospitals contributing consecutive data from 2004 to 2012 were included in the trend analysis (n = 33). Similar models were used to examine the use of specific modalities (i.e., ECMO and RRT) over time and to compare odds of mortality among subgroups of patients with PSS using ET.

## Results

### Utilization rate of extracorporeal therapies

In total, 636,482 PICU admissions were reported from the 43 hospitals after excluding 2269 patients who had a concurrent NICU admission during the same hospitalization. Among the PICU patients, 7.7 % (49,153 patients) met our definition of PSS. This PSS cohort was analyzed further for ET utilization. In this group, 4795 (9.8 %) patients received at least one form of ET. RRT was the more commonly used modality (7.0 %), followed by ECMO (3.8 %) (Table 1). Among patients receiving ET, few received both ET modalities (500 patients, 1.0 %) (Table 2).

**Table 1:** Demographics of patients with pediatric severe sepsis on extracorporeal therapy

All patients from 2004 to 2012	Any ET	ECMO	RRT
N = 49,153 PSS cases			
Use in PSS, N (%)	4795 (9.8 %)	1858 (3.8 %)	3437 (7.0 %)
Gender, N (%)			
Male	2452 (51.2 %)	978 (52.7 %)	1728 (50.3 %)
Female	2342 (48.8 %)	879 (47.3 %)	1709 (49.7 %)
Age group at admission, N (%)			
<1 year	1198 (25.0 %)	830 (44.7 %)	537 (15.6 %)
1–4 years	1102 (23.0 %)	421 (22.7 %)	783 (22.8 %)
5–9 years	657 (13.7 %)	192 (10.3 %)	531 (15.5 %)
10–18 years	1838 (38.3 %)	415 (22.3 %)	1586 (46.1 %)
Total hospitalization, days			
Median (25 %–75 %)	34 (17–64)	35 (16–67)	34 (17–62)
ICU, days			
Median (25 %–75 %)	19 (8–38)	25 (12–48)	17 (7–34)
Co-morbidities*,* N (%)			
Any condition	3745 (78.1 %)	1,456 (78.4 %)	2645 (77.0 %)
Neurologic	580 (12.1 %)	198 (10.7 %)	424 (12.3 %)
Cardiovascular	1734 (36.2 %)	1119 (60.2 %)	849 (24.7 %)
Respiratory	263 (5.5 %)	156 (8.4 %)	141 (4.1 %)
Renal	767 (16.0 %)	92 (5.0 %)	708 (20.6 %)
Gastroenterology	304 (6.3 %)	64 (3.4 %)	258 (7.5 %)
Hematology/Immunology	452 (9.4 %)	139 (7.5 %)	347 (10.1 %)
Metabolic disorder	976 (20.4 %)	207 (11.1 %)	844 (24.6 %)
Malignancy	855 (17.8 %)	133 (7.1 %)	773 (22.5 %)
Other	417 (8.7 %)	216 (11.6 %)	234 (6.8 %)
Acute organ dysfunction			
Number of systems affected, N (%)			
1	969 (20.2 %)	286 (15.4 %)	728 (21.2 %)
2	1779 (37.1 %)	831 (44.7 %)	1133 (33.0 %)
3	1187 (24.8 %)	462 (24.9 %)	878 (25.6 %)
4	611 (12.7 %)	205 (11.0 %)	494 (14.4 %)
5+	249 (5.2 %)	74 (4.0 %)	204 (5.9 %)

*PSS* pediatric severe sepsis, *ET* extracorporeal therapy, *ECMO* extracorporeal membrane oxygenation, *RRT* Renal replacement therapy

**Table 2:** Extracorporeal therapy use by modality

All patients from 2004 to 2012	ECMO-only (N = 1,358)	RRT-only (N = 2,937)	ECMO + RRT (N = 500)
Prevalence (N = 49,153)	1358 (2.3 %)	2937 (6.0 %)	500 (1.0 %)
Gender, N (%)			
Male	724 (53.4 %)	1474 (50.2 %)	254 (50.8 %)
Female	633 (46.6 %)	1463 (49.8 %)	246 (49.2 %)
Mortality, N (%)	649 (47.8 %)	948 (32.3 %)	290 (58.0 %)
Age group at admission	1.1 (0.2–6.7)	9.6 (2.6–14.8)	3.9 (0.4–12.8)
Median (25 %–75 %)			
Total hospitalization, days	34 (17–69)	33 (17–62)	36 (16–60)
Median (25 %–75 %)			
ICU, days	24 (12–49)	16 (6–32)	27 (12–46)
Median (25 %–75 %)			

*ECMO* extracorporeal membrane oxygenation, *RRT* Renal replacement therapy, *ICU* intensive care unit

ET utilization was higher in PSS patients with multiple organ dysfunction syndrome (MODS) defined as OD of at least 3. Among these patients (8310 patients, 16.9 % of the PSS cohort), 2047 (24.6 %) received at least one ET modality. ECMO use was seen in 8.9 % of PSS patients with MODS, whereas RRT was used in 19.0 % of this patient population.

### Clinical demographics

Overall, ET use in patients with PSS was divided equally among gender; 51 % of those receiving any ET were male and 49 % were female. Patients less than 1 year of age accounted for the majority of those receiving only ECMO (48.7 %) (Table 3). This subgroup also accounted for the majority of patients receiving both modalities, representing 169 (33.8 %) of patients who received both ECMO + RRT.

**Table 3:** Prevalence of extracorporeal therapy use and mortality by age, co-morbidity, and organ dysfunction

Characteristic	ECMO alone (N = 1358)		RRT alone (N = 2937)		ECMO + RRT (N = 500)	
	Prevalence	Mortality	Prevalence	Mortality	Prevalence	Mortality
Age						
<1 year	661 (48.7 %)	344 (52.0 %)	368 (12.5 %)	144 (39.1 %)	169 (33.8 %)	111 (65.7 %)
1-4 years	319 (23.5 %)	130 (40.8 %)	681 (23.2 %)	197 (28.9 %)	102 (20.4 %)	61 (59.8 %)
5-9 years	126 (9.3 %)	57 (45.2 %)	465 (15.8 %)	133 (28.6 %)	66 (13.2 %)	27 (40.9 %)
10-18 years	252 (18.6 %)	118 (46.8 %)	1423 (48.5 %)	474 (33.3 %)	163 (32.6 %)	91 (55.8 %)
Co-morbidity						
Any condition	1100 (81.0 %)	522 (47.5 %)	2289 (77.9 %)	806 (35.2 %)	356 (71.2 %)	206 (71.0 %)
Neurologic	156 (11.5 %)	57 (36.5 %)	382 (13.0 %)	113 (29.6 %)	42 (8.4 %)	21 (50.0 %)
Cardiovascular	885 (65.2 %)	404 (45.7 %)	615 (20.9 %)	261 (42.4 %)	234 (46.8 %)	135 (57.7 %)
Respiratory	122 (9.0 %)	50 (41.0 %)	107 (3.6 %)	52 (48.6 %)	34 (6.8 %)	25 (73.5 %)
Renal	59 (4.3 %)	31 (52.5 %)	675 (23.0 %)	129 (13.6 %)	33 (6.6 %)	18 (54.6 %)
Gastroenterology	46 (3.4 %)	23 (50.0 %)	240 (8.2 %)	107 (44.6 %)	18 (3.6 %)	13 (72.2 %)
Hematology/Immunology	105 (7.7 %)	57 (54.3 %)	313 (10.7 %)	144 (15.2 %)	34 (6.8 %)	21 (61.8 %)
Metabolic disorder	132 (9.7 %)	61 (46.2 %)	769 (26.2 %)	210 (27.3 %)	75 (15.0 %)	38 (50.7 %)
Malignancy	82 (6.0 %)	52 (63.4 %)	723 (24.6 %)	381 (52.7 %)	50 (10.0 %)	29 (58.0 %)
Other	183 (13.5 %)	94 (51.4 %)	201 (6.8 %)	63 (31.3 %)	33 (6.6 %)	22 (66.7 %)
None	258 (19.0 %)	127 (49.2 %)	648 (22.1 %)	142 (21.9 %)	144 (28.8 %)	84 (58.3 %)
Organ dysfunction						
1	241 (17.8 %)	122 (50.6 %)	683 (23.3 %)	82 (12.0 %)	45 (9.0 %)	27 (60.0 %)
2	646 (47.6 %)	264 (40.9 %)	948 (32.3 %)	299 (31.5 %)	185 (37.0 %)	102 (55.1 %)
3	309 (22.8 %)	163 (52.8 %)	725 (24.7 %)	302 (41.7 %)	153 (30.6 %)	89 (58.2 %)
4	117 (8.6 %)	72 (61.5 %)	406 (13.8 %)	182 (44.8 %)	88 (17.6 %)	52 (59.1 %)
5+	45 (3.3 %)	28 (62.2 %)	175 (6.0 %)	83 (47.4 %)	29 (5.8 %)	20 (69.0 %)

*ECMO* extracorporeal membrane oxygenation, *RRT* Renal replacement therapy

RRT alone was most common in children 10–18 years old (48.5 %). Patients who received any form of ET had a prolonged hospitalization with median hospital LOS (length of stay) of 34 days (IQR of 17–64 days) and PICU LOS of 19 days (IQR of 8–38 days) (Table 1).

### Co-morbidity and organ dysfunction

A significant proportion of PSS patients receiving ET have at least one underlying co-morbidity (78.1 %) compared with the baseline of 73.8 % in the general PSS cohort [2]. Underlying cardiovascular dysfunction was the most common co-morbidity found in patients receiving ECMO alone (65.2 %). A cardiovascular co-morbidity also was the most common underlying condition in those receiving combination therapies (46.8 % of those receiving ECMO + RRT). The most common co-morbidities found in patients receiving RRT alone were metabolic disorder (26.2 %) and malignancy (24.6 %), ahead of renal causes (23.0 %) (Table 3).

The majority of PSS patients who were placed on ET had at least two organs with dysfunction (OD), accounting for 79.8 % patients of PSS patients on ET. ET use was associated with the presence of MODS. MODS was present in 42.7 % of PSS patients receiving ET but in only 14.1 % of PSS patients who did not receive ET.

### Mortality

Overall mortality rate in PSS patients who received ET was 39.4 % (1887 patients). Mortality rates were 47.8 % in PSS patients receiving ECMO alone (n = 649), 32.3 % in those receiving RRT alone (n = 2937), and 58.0 % in those receiving both ECMO and RRT (n = 500) (Table 2). Among PSS patients receiving ECMO alone, patients less than 1 year of age had the highest rate of mortality (344 out of 661 patients, or 52.0 %). In patients receiving only RRT, mortality was also highest in those less than 1 year of age (144 out of 368 patients, or 39.1 %) (Table 3).

Patients with malignancy had the highest mortality rate (462 patients, or 54.0 %) out of those who received any form of ET, ECMO alone (52 out of 82 patients, or 63.4 %), and RRT alone (381 out of 723 patients, or 52.7 %).

PSS patients under 1 year of age represented the largest proportion of patients receiving multiple ET modalities: they account for 33.8 % of patients who received ECMO + RRT (169 out of 500 patients). They also had the highest mortality rate (65.7 %). In patients with MODS receiving ET, mortality was high (48.4 %). Mortality was higher in patients receiving ECMO (47.8 %) compared with those on RRT (32.3 %) (*P* < 0.001). Odds of mortality increased sequentially with increasing OD. PSS patients on ET with severe OD (≥5) had higher odds of mortality compared with patients on ET with one OD (OR = 3.60, 95 % CI 2.68–4.84, *P* <0.001). The highest mortality rate in patients with MODS was among those who received both ET modalities (161 out of 270, or 59.6 %).

### Trends

**Fig. 1:**
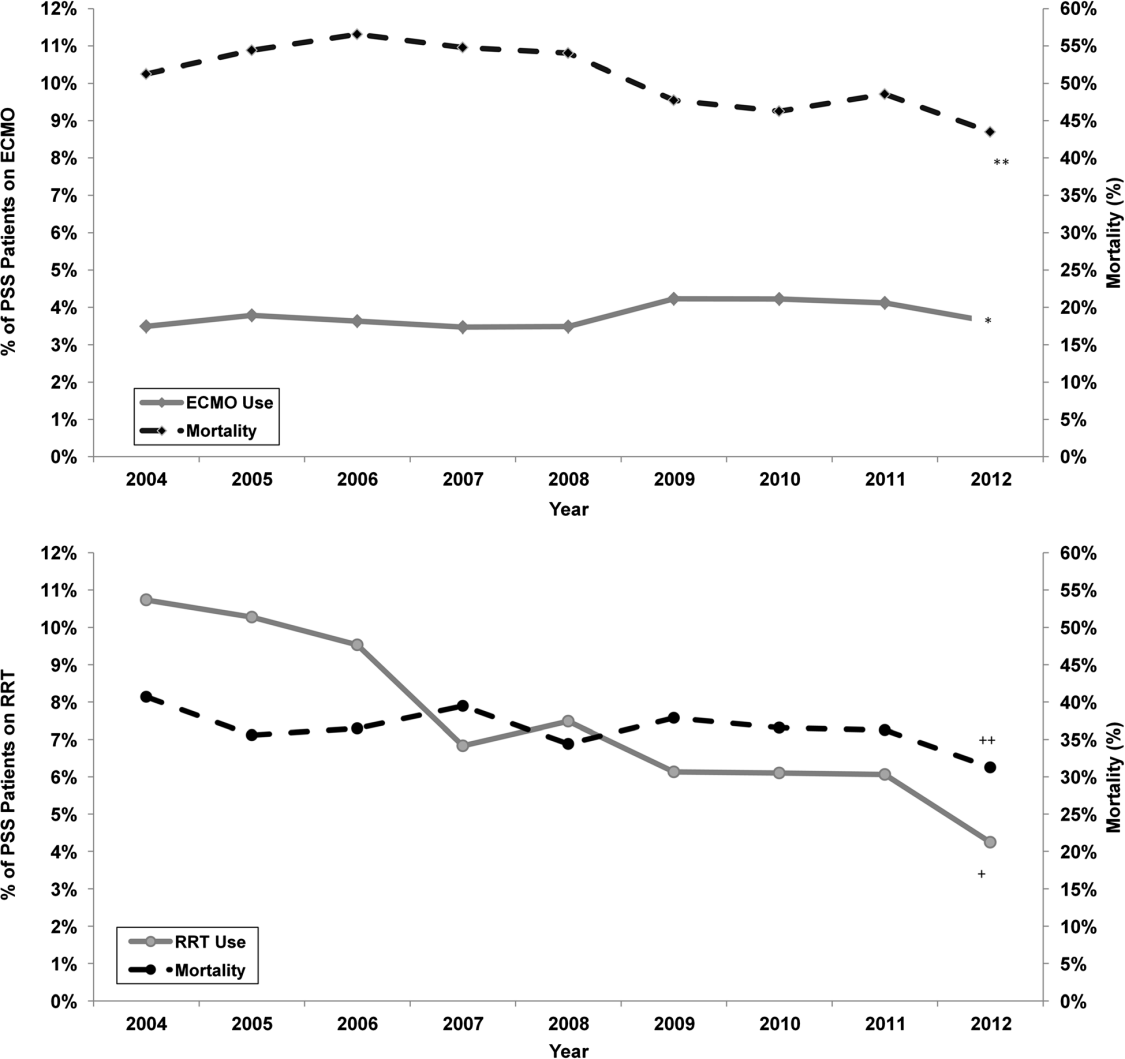
*Top*: the *solid line* represents the prevalence of ECMO use in the patients with PSS. The *broken line* represents mortality rate in PSS patients who received ECMO. *indicates a significant increase in the prevalence of patients receiving ECMO. **indicates a significant decrease in mortality over time. *Bottom*: the *solid line* represents RRT use in the overall PSS patient population. The *broken line* represents mortality rate over time in the patients with PSS. *ECMO* extracorporeal membrane oxygenation, *PSS* pediatric severe sepsis, *RRT* Renal replacement therapy

ET usage overall in patients with PSS significantly decreased over time (13.3 % in 2004 % to 7 % in 2012 %, *P* < 0.001); however, patterns of use for specific modalities over time are not consistent. Across all patients, the rate of ECMO utilization remained steady from 2004 to 2008 but showed moderate increases from 2008 to 2012. After adjustment for cases from the same hospital and severity of OD, patients with PSS from 2009 to 2012 had higher odds of receiving ECMO compared with patients from 2004 to 2008 (OR = 1.18, 95 % CI 1.06–1.33, *P* = 0.004). In contrast, there was a significant decrease in RRT over time (Fig. 1, *P* < 0.001). We performed additional analysis to determine the impact of specific co-morbidities. ECMO utilization significantly increased in PSS patients with malignancy co-morbidities over the 9-year period (4.5 % in 2004 to 9.0 % in 2012). However, after removal of this small number of patients with malignancies, the odds of receiving ECMO for patients with PSS remained unchanged and was still higher for patients in the 2009–2012 versus the 2004–2008 time periods (OR = 1.18, 95 % CI 1.05–1.33).

Patients with PSS from 2009 to 2012 had lower odds of receiving RRT compared with patients from 2004 to 2009 (OR = 0.64, 95 % CI 0.59–0.69, *P* < 0.001). Excluding the malignancy population changed the odds to 0.60 (95 % CI 0.55–0.66, *P* < 0.001).

**Fig. 2:**
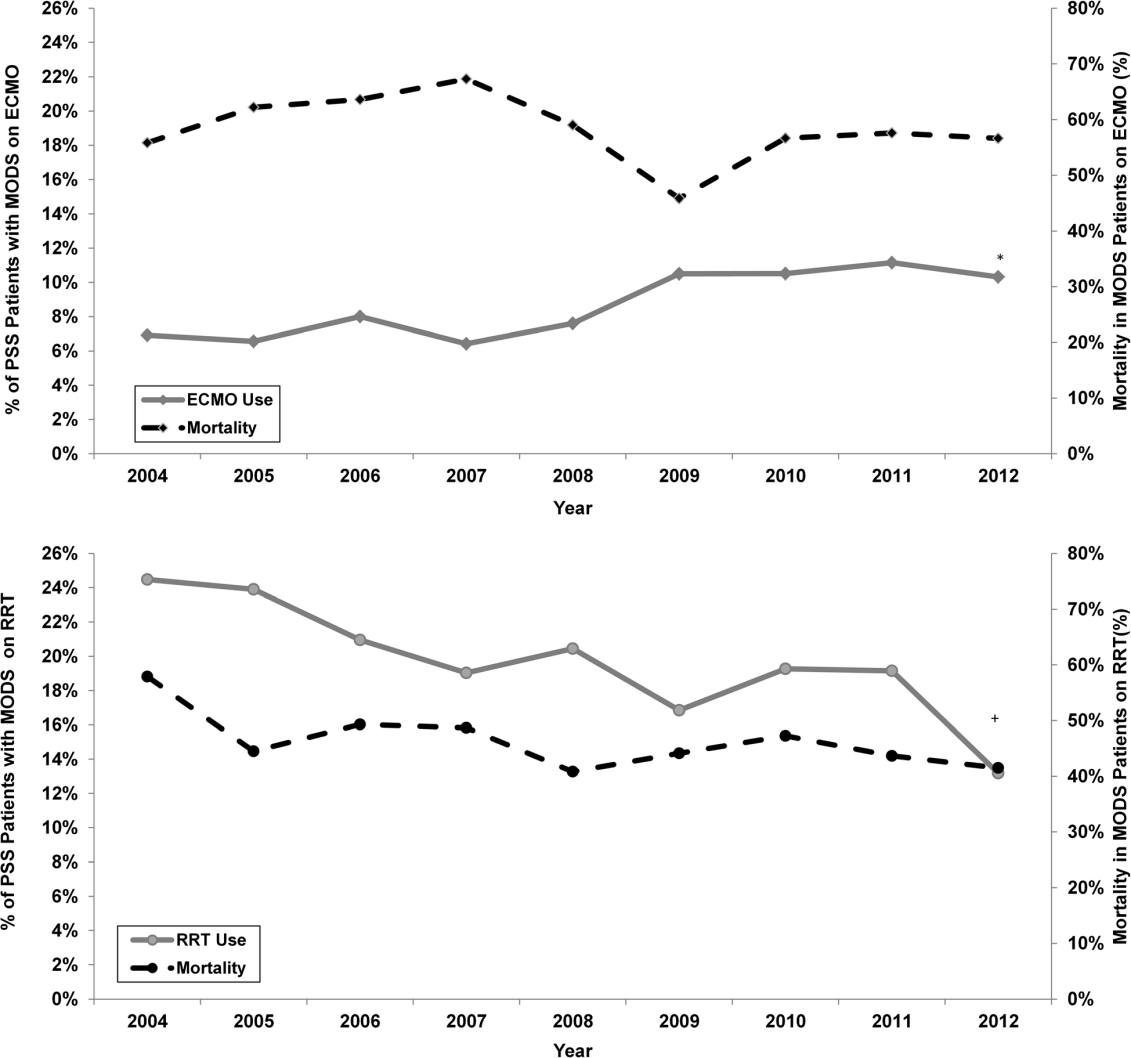
*Top*: the *solid line* represents the prevalence of ECMO use in PSS patients with MODS. The *broken line* represents the mortality rate of MODS patients on ECMO. *indicates a significant increase in the utilization of ECMO in PSS patients with MODS. *Bottom*: the *solid line* represents the prevalence of RRT use in PSS patients with MODS. The *broken line* represents mortality rate in MODS patients who received RRT. *ECMO* extracorporeal membrane oxygenation, *MODS* multiple organ dysfunction syndrome, *PSS* pediatric severe sepsis, *RRT* Renal replacement therapy

When RRT use in patients with MODS was examined (Fig. 2), rates also significantly declined (*P* < 0.001) during the same time intervals. Odds of receiving RRT in PSS MODS patients decreased by 27 % from 2009 to 2012 compared with 2004 to 2008 (OR = 0.72, 95 % CI 0.63– 0.83). In contrast, ECMO rates increased over time (Fig. 2, *P* = 0.001), and the odds of receiving ECMO in PSS patients with MODS was 1.5 times higher from 2009 to 2012 compared with 2004 to 2008 (95 % CI 1.24–1.80, *P* <0.001).

In this cohort of PSS patients on ET, yearly mortality rates showed small decreases over time (41.9 % in 2004 versus 36.3 % in 2012); however, odds of mortality decreased by only 6 % per year (OR = 0.94, 95 % CI 0.93–0.95, *P* < 0.001). A similar trend was observed in mortality in PSS patients specifically receiving ECMO (Fig. 1). Odds of mortality decreased by 7 % per year (OR = 0.93, 95 % CI 0.90–0.97, *P* < 0.001). For PSS patients with malignancy on ECMO, the mortality odds had a more substantial drop of 21 % per year (OR = 0.79, 95 % CI 0.66–0.95, *P* = 0.011).

A similar, but less pronounced, trend in mortality was observed in overall PSS patients receiving RRT with odds of mortality decreasing by 4 % per year (OR = 0.96, 95 % CI (0.93–0.99, *P* = 0.008). In PSS patients with malignancy receiving RRT (n = 648), the odds significantly decreased over time (OR = 0.90, 95 % CI 0.84–0.95, *P* < 0.001). Overall in PSS patients with MODS on ET, mortality also decreased from 2004 to 2012 (56.8 % to 48.5 %), but odds of mortality decreased by only 4 % per year (OR = 0.96, 95 % CI 0.93–0.999, *P* = 0.0439).

In patients receiving ET in our 10-year cohort (n = 4795), median (25 %–75 %) hospitalization cost was $270,778 ($122,506–$528,732) and significantly higher compared with median cost of $69,070 ($29,931–$155,864) in PSS patients who did not receive any ET. When accounting for LOS, median cost per day was 1.8 times higher in patients receiving ET compared with those who did not ($7,591 versus $4,334). In hospitals with data over the entire 10-year period for PSS (n = 33 hospitals), there was a significant negative relationship between center volume and mortality for patients on ECMO (r_s_ = −0.43, 95 % CI −0.67 to −0.09, *P* = 0.013); that is, mortality decreased with increasing center volume of ECMO cases.

## Discussion

In this largest reported analysis of ET utilization in PSS to date, overall rates were relatively consistent throughout the time period studied. However, we did find that in a time period coinciding with publication of the 2009 ACCM guidelines for severe sepsis management with revised recommendations of ECMO consideration for refractory PSS, ECMO utilization significantly increased, especially in PSS patients with MODS (10 % from 2009 to 2012 versus 7 % in 2004 to 2008).

Excluding primary NICU patients, children under 1 year old continue to represent a disproportionately large percentage of those receiving ECMO. Although our data did not allow us to further analyze the root cause of this, possible explanations include the prevalence of congenital conditions (i.e., congenital heart disease, neurological malformation, etc.) in this population and history of prematurity with multiple accompanying medical conditions. Associated cardiovascular co-morbidity was found in a large percentage of all patients receiving ECMO and represents a significant proportion of PSS patients receiving multiple modalities. This may be because ECMO is used more commonly in cardiac patients compared with general medical patients. As might be expected, degree of OD impacted use of ET. Children with at least three ODs had a significantly higher proportion of ET utilization than those with fewer ODs. This held true when analyzing those who received ECMO and RRT separately (34.7 % of those receiving ECMO, 44.5 % of those receiving RRT, and 54.0 % receiving both had at least three ODs).

We previously reported that overall PSS mortality has been declining with increasing prevalence since 2004 [2]. Mortality was high in PSS patients who received ET, reflective of the greater severity of illness of these patients. The overall mortality of PSS patients on RRT was 32.3 % in our study. The overall ECMO mortality rate of 47.8 % is comparable to recently published reports of a 40 % mortality rate in ECMO patients with acute respiratory distress syndrome secondary to sepsis [21]. For PSS patients receiving both RRT and ECMO, the mortality was even higher at 60 %. Despite these high rates, we found a significant improvement over time in overall survival in patients receiving ECMO. This was more pronounced in the population of PSS patients with malignancy, whose mortality odds went down substantially over the periods of the study. Similar trends were seen in mortality rates for malignancy patients receiving RRT, with lower mortality odds over time as well.

The improvement in survival rate was associated with a tendency for improved outcomes in higher-volume ECMO centers. Improved outcomes in ECMO and RRT patients with malignancy are in line with reports of improved outcomes on ECMO with conditions such as cancer previously thought to be contraindications to ECMO use [22].

Our study has several limitations in addition to its respective nature. The administrative nature of the PHIS database, which has billing and resource utilization data but is missing some important clinical parameters, limits the ability to make better clinical correlations. Thus, we were unable to assess some variables that may have been clinically significant, such as whether a patient was placed on veno-arterial versus veno-venous ECMO. Another major limitation is the lack of variables needed to calculate a severity-of-illness score. While we used OD as a surrogate, detailed clinical data to calculate PRISM-III (The Pediatric Risk of Mortality III) or PELOD (Pediatric Logistic Organ Dysfunction) scores are not available in PHIS. Because data are derived from ICD-9 codes collected throughout hospitalization, we were also unable to determine whether death occurred while a patient was undergoing an ET or afterwards. Hospitals in the PHIS database are generally tertiary or quaternary referral centers and treat more medically complex patients than other community-based pediatric facilities that are not represented in PHIS. This may limit generalization of conclusions drawn from studying PHIS centers. Finally, reported mortality reported is all-cause mortality. A patient who survived the initial sepsis episode could have expired from another cause later during the hospitalization, but the data do not allow one to distinguish between causes of death. This limits absolute conclusions regarding mortality and ET use.

## Conclusions

In this large multicenter PSS PICU cohort, ECMO use in PSS significantly increased since 2009. There was a similarly significant increase in ECMO utilization after 2009 in patients with MODS. This may have been related to increasing support in the literature on successful use of ECMO in patients with co-morbidities as well as recommendations in the ACCM sepsis guidelines supporting ECMO consideration. Mortality rates in PSS patients receiving ECMO decreased over time and are comparable to the latest nationally published pediatric sepsis outcome data. In PSS patients with malignancy, there is a statistically significant improvement in survival rate for those placed on ECMO and RRT. Further research with more clinically robust data is needed to determine the impact of different ET modalities in the PSS patient population.

## Key messages

- PSS remains a relevant health issue in the US pediatric population. ET utilization in this cohort has not been adequately described.
- ECMO and RRT were used in a small percentage of children with PSS, with a higher rate of utilization in PSS patients with MODS.
- From 2004 through 2012, overall utilization of ET in patients with PSS remained relatively unchanged. However, ECMO use significantly increased after 2009, especially in patients with MODS.
- Mortality rates in PSS patients receiving ECMO and RRT were consistent with previously published reports; survival improved over the time period in PSS patients receiving ECMO.
- ET can provide potentially beneficial adjunctive treatment in patients with PSS. Further research and clinical correlation to these epidemiologic findings are warranted.

## Additional file

Additional file 1:
**List of participating Children’s Hospital Association institutions.** (DOC 52 kb)

## Abbreviations

ACCM: American College of Critical Medicine
CHA: Children’s Hospital Association
CI: Confidence interval
CTC: Clinical transaction code
ECMO: Extracorporeal membrane oxygenation
ET: Extracorporeal therapy
ICD-9: International Classification of Diseases, 9th Revision
IQR: Interquartile range
LOS: Length of stay
MODS: Multiple organ dysfunction syndrome
NICU: Neonatal intensive care unit
OD: Organ dysfunction
OR: Odds ratio
PHIS: Pediatric Health Information System
PICU: Pediatric Intensive Care Unit
PSS: Pediatric severe sepsis
RRT: Renal replacement therapy
