# Supplementary material for: Extracorporeal therapies in pediatric severe sepsis: findings from the pediatric health-care information system
Source: Crit Care. 2015 Nov 10;19:397. doi: 10.1186/s13054-015-1105-4 (PMC4640405; doi:10.1186/s13054-015-1105-4)
Supplement: Additional file 1: — List of participating Children’s Hospital Association institutions. (DOC 52 kb) [file 13054_2015_1105_MOESM1_ESM.doc]

**List of participating Children's Hospital Association institutions**

| Rady Children's Hospital San Diego* | San Diego, California |
| --- | --- |
| Connecticut Children's Medical Center | Hartford, Connecticut |
| Children’s Hospital Colorado* | Denver, Colorado |
| Lucile Packard Children's Hospital at Stanford | Palo Alto, California |
| Children’s Mercy Hospitals and Clinics* | Kansas City, Missouri |
| Riley Hospital for Children at Indiana University Health* | Indianapolis, Indiana |
| Le Bonheur Children's Medical Center* | Memphis, Tennessee |
| East Tennessee Children's Hospital | Knoxville, Tennessee |
| Children's National Medical Center* | Washington, DC |
| Primary Children's Medical Center | Salt Lake City, Utah |
| Children’s Hospital of Pittsburgh of UPMC* | Pittsburgh, Pennsylvania |
| Children’s Hospital & Research Center Oakland* | Oakland, California |
| Ann & Robert H. Lurie Children's Hospital of Chicago* | Chicago, Illinois |
| Phoenix Children's Hospital | Phoenix, Arizona |
| Akron Children’s Hospital* | Akron, Ohio |
| Boston Children’s Hospital | Boston, Massachusetts |
| The Children's Medical Center of Dayton | Dayton, Ohio |
| Women and Children’s Hospital of Buffalo | Buffalo, New York |
| Children’s Hospital Central California* | Fresno, California |
| Monroe Carell Jr. Children's Hospital at Vanderbilt* | Nashville, Tennessee |
| Children’s Hospital of The King’s Daughters | Norfolk, Virginia |
| New York-Presbyterian/Morgan Stanley Children’s Hospital* | New York, New York |
| All Children's Hospital* | St. Petersburg, Florida |
| Children’s Medical Center | Dallas, Texas |
| Arkansas Children's Hospital* | Little Rock, Arkansas |
| The Children's Hospital of Philadelphia* | Philadelphia, Pennsylvania |
| Children's Hospital of Orange County* | Orange, California |
| Children’s Hospitals and Clinics of Minnesota* | St. Paul/Minneapolis, Minnesota |
| Driscoll Children's Hospital* | Corpus Christi, Texas |
| Children’s Hospital of Michigan* | Detroit, Michigan |
| Miami Children's Hospital* | Miami, Florida |
| Nationwide Children’s Hospital* | Columbus, Ohio |
| Cook Children’s Health Care System* | Fort Worth,Texas |
| Children's Hospital* | New Orleans, Louisiana |
| Children’s of Alabama* | Birmingham, Alabama |
| Children’s Hospital & Medical Center* | Omaha, Nebraska |
| Children's Hospital of Wisconsin* | Milwaukee, Wisconsin |
| Texas Children's Hospital* | Houston, Texas |
| Cincinnati Children's Hospital Medical Center* | Cincinnati, Ohio |
| St. Louis Children's Hospital* | St. Louis, Missouri |
| Children’s Hospital Los Angeles | Los Angeles, California |
| Children's Healthcare of Atlanta* | Atlanta, Georgia |
| Seattle Children’s Hospital* | Seattle, Washington |

*denotes hospital with continuous data utilized for trend analysis
